# The Role of Lurasidone in Managing Depressive Symptoms in People with Schizophrenia: A Review

**DOI:** 10.3390/brainsci14030225

**Published:** 2024-02-28

**Authors:** Andrea Fiorillo, Gaia Sampogna, Umberto Albert, Emi Bondi, Serafino De Giorgi, Andrea Fagiolini, Maurizio Pompili, Gianluca Serafini, Umberto Volpe, Antonio Vita

**Affiliations:** 1 Department of Psychiatry, University of Campania “L. Vanvitelli”, 80138 Naples, Italy; 2 Department of Medicine, Surgery and Health Sciences, University of Trieste, 34127 Trieste, Italy; 3 Department of Mental Health, Azienda Sanitaria Universitaria Giuliano Isontina-ASUGI, 34127 Trieste, Italy; 4 Department of Mental Health and Addictions, ASST Papa Giovanni XXIII, 24121 Bergamo, Italy; 5 Department of Mental Health, ASL Lecce, 73100 Lecce, Italy; 6 Division of Psychiatry, University of Siena, 53100 Siena, Italy; 7 Department of Neurosciences, Mental Health and Sensory Organs, Sant’Andrea Hospital, Sapienza University of Rome, Via di Grottarossa, 1035, 00189 Rome, Italy; 8 Section of Psychiatry, Department of Neuroscience, Rehabilitation, Ophthalmology, Genetics, Maternal and Child Health (DINOGMI), University of Genoa, 16131 Genoa, Italy; 9 IRCCS Ospedale Policlinico San Martino, 16131 Genoa, Italy; 10 Clinical Psychiatry Unit, Department of Clinical Neurosciences, Università Politecnica delle Marche, 60126 Ancona, Italy; 11 Department of Clinical and Experimental Sciences, University of Brescia, 25123 Brescia, Italy; antonio.vita@unibs.it

**Keywords:** schizophrenia, personalized treatment, depressive symptoms, antipsychotic, lurasidone

## Abstract

Background: Schizophrenia is a severe mental disorder characterized by positive, negative, affective, and cognitive symptoms. Affective symptoms in patients with schizophrenia have traditionally been overlooked or even neglected because they are not considered as fundamental as positive and negative symptoms in the choice of medication. Methods: This paper aims to systematically evaluate the efficacy and safety of lurasidone in the treatment of depressive symptoms of schizophrenia. Results: Lurasidone appears to be particularly effective on the depressive symptomatology of schizophrenia while also alleviating the positive and negative symptoms associated with the illness. Conclusions: The efficacy of lurasidone in treating patients with first-episode psychosis who present with predominant depressive symptoms suggests that this medication may be a valuable treatment option not only for established cases of schizophrenia but also for individuals in the early stages of the illness. The good tolerability of lurasidone is an important factor that may positively influence treatment decisions.

## 1. Introduction

Schizophrenia is a severe mental disorder with a heterogeneous clinical presentation, characterized by different symptomatologic clusters named positive, negative, affective, and cognitive domains [1]. There is considerable overlap between these different symptom domains, and the distinction between negative and affective symptoms (including depression and anxiety) can sometimes be clinically difficult. Both domains can negatively impact patients’ level of functioning and quality of life; therefore, it is mandatory to early detect those symptoms and develop a targeted, individualized treatment plan for each patient [2].

Depressive symptoms in patients with schizophrenia have traditionally been overlooked or even neglected because they are not considered as fundamental as positive and negative symptoms in the choice of medication (typically, first-generation antipsychotics are more effective for positive symptoms, while second-generation antipsychotics are more effective for negative symptoms).

Due to the increasing prevalence of the Kraepelin dichotomy (i.e., distinction between psychotic “manic-depression” and “dementia praecox”) in current diagnostic manuals and guidelines [3,4,5], as well as the dominance of the categorical diagnostic approach in clinical psychiatry [6,7,8], the relationship between psychotic and depressive symptoms of schizophrenia remains a diagnostic conundrum whose boundaries still seem unclear to many clinicians and researchers [9,10,11,12].

Several recent studies have attempted to clarify the predictive and prognostic role of depressive symptoms in patients with schizophrenia. The available data seem quite controversial, with some authors highlighting that the presence of depressive symptoms is associated with a good prognosis [13], while other studies have found a negative association between depressive symptoms and outcome in schizophrenia. In particular, several authors have reported that psychotic patients with depressive symptoms have earlier relapses, more frequent and longer hospitalizations, a poorer prognosis, and a worse outcome [14,15,16].

A higher prevalence rate of depression has been found in patients with schizophrenia compared with the general population, with estimates ranging from 7 to 65%. Patients with schizophrenia with a comorbid depressive disorder report poor quality of life, increased suicidal risk, poor medication adherence, higher rates of relapse and hospitalization, poor physical health, and worse long-term outcomes compared to people with schizophrenia without depressive symptoms.

More recently, studies focusing on the clinical high-risk and ultra-high-risk populations have found that patients with high sensitivity to stress and a tendency to develop negative depressive states are at higher risk of developing full-blown psychotic disorders [17,18,19]. In addition, experience sampling studies have found that patients with schizophrenia spectrum disorders exhibit more negative affect in their daily lives, while laboratory studies have shown that these patients have a bias toward judging neutral stimuli as more negative.

Negative affect in patients with schizophrenia predicts poorer functional outcomes, more hospitalizations, reduced quality of life, increased need for psychiatric treatment, and more frequent suicide, even after controlling for negative, neurocognitive, and positive symptoms [20].

Negative symptoms of schizophrenia also play a critical role in determining poor outcomes, as they contribute significantly to poor psychosocial functioning and quality of life, are long-lasting, and place a significant burden on caregivers [21]. In addition, these symptoms decrease patients’ motivation to seek treatment and reduce treatment adherence [22]. As a result, people with schizophrenia are often unemployed, single, poorly educated, and have few social contacts, which is reflected in their everyday difficulties. Their family members often feel stigmatized and may be exposed to social isolation [23].

With new treatment approaches, the improved safety of modern antipsychotics, and the fulfillment of some of the unmet needs, the outcome of patients with schizophrenia is changing, and many can now achieve full recovery. This should be the goal of clinicians dealing with schizophrenia [24]. The so-called third-generation antipsychotics, which include cariprazine, lurasidone, and brexpiprazole, have recently been introduced as monotherapy or adjuncts for the treatment of depressive and negative symptoms in patients with schizophrenia [25,26,27,28]. Available studies suggest that they are particularly useful for these symptoms, have a relatively safe tolerability profile, and are well perceived by patients [29,30,31,32]. In this paper, we aim to review the efficacy of lurasidone for the depressive symptoms of schizophrenia and try to identify the benefits and pitfalls of using lurasidone in clinical practice. Lurasidone is a benzisothiazole derivative approved by the US FDA for the treatment of schizophrenia, major depression, and depressive episodes associated with bipolar I disorder. Lurasidone is also approved by the EMA for the treatment of schizophrenia in adults and by Health Canada for the treatment of acute cases of schizophrenia and bipolar depression. Functionally, lurasidone is primarily an antagonist of D2 and 5-HT2A receptors. The D2 antagonism is associated with its efficacy in the treatment of schizophrenia, while the potent 5-HT2A antagonism improves the negative symptoms of schizophrenia and reduces extrapyramidal side effects and prolactin levels. In addition, lurasidone’s unique profile includes potent 5-HT7 receptor antagonism associated with cognitive enhancement. Lurasidone’s 5-HT1A receptor partial agonism explains its anxiolytic and antidepressant effects; the drug’s binding affinity and antagonism at noradrenergic a2C receptors improve cognitive and motor function. Another positive aspect of lurasidone is its lack of antimuscarinic and anti-H1 histamine activity, which further reduces its side effects. Adverse effects such as akathisia, somnolence, and prolactin elevation are generally manageable with dose adjustment, and its modest noradrenergic alpha-1 antagonist activity (more than 20-fold less potent than its binding affinities at D2 and 5-HT2A receptors) implies a lower risk of postural hypotension and tachycardia. The pharmacodynamic profile of lurasidone is consistent with its efficacy in treating positive, negative, and depressive symptoms of schizophrenia while maintaining a relatively favorable tolerability profile. Nevertheless, the focus of research and attention on antipsychotics has been predominantly on their efficacy in treating positive and negative symptoms. In light of this, we conducted a comprehensive review of the available literature to systematically assess the efficacy of lurasidone in the treatment of depressive symptoms associated with schizophrenia.

## 2. Materials and Methods

Clinical trials evaluating the use of lurasidone for the treatment of depressive disorders in patients with schizophrenia spectrum disorders were identified using the following search terms: “schizophrenia”, “schizoaffective disorder”, “psychosis”, “first episode of psychosis”, “depressive symptoms”, “affective symptoms”, “pharmacological treatment”, “atypical antipsychotics”, “second-generation antipsychotics”, and “lurasidone”. Keywords were entered into PubMed, ISI Web of Knowledge, Scopus, and Medline databases and combined in order to identify relevant papers by making the search more restrictive or detailed.

The search strategy was limited to the period from inception to 31 December 2022.

The following criteria have been considered: (1) studies including patients with a diagnosis of schizophrenia, schizoaffective, or psychotic disorder; (2) the antipsychotic pharmacological treatment included lurasidone; (3) the primary or secondary outcomes considered in the study included affective/depressive symptoms. 

Papers were excluded when antipsychotic pharmacological treatments included drugs different from lurasidone. Studies focused only on the efficacy of lurasidone on positive or negative symptoms without specific data on depressive/affective symptoms were excluded.

The following study designs were included: case reports/case series, open label studies, case/control studies, and randomized controlled trials (RCTs). Non-original research, such as systematic reviews, meta-analyses, and narrative reviews, was excluded.

All selected papers were evaluated by two researchers (GS and AF). After evaluating the abstracts, the main data from the included papers were extracted: (1) authors and country; (2) study design; (3) sample size; (4) assessment tools; (5) main findings; (6) study limitations.

The systematic review has been conducted according to the PRISMA guidelines.

## 3. Results

**Figure 1:**
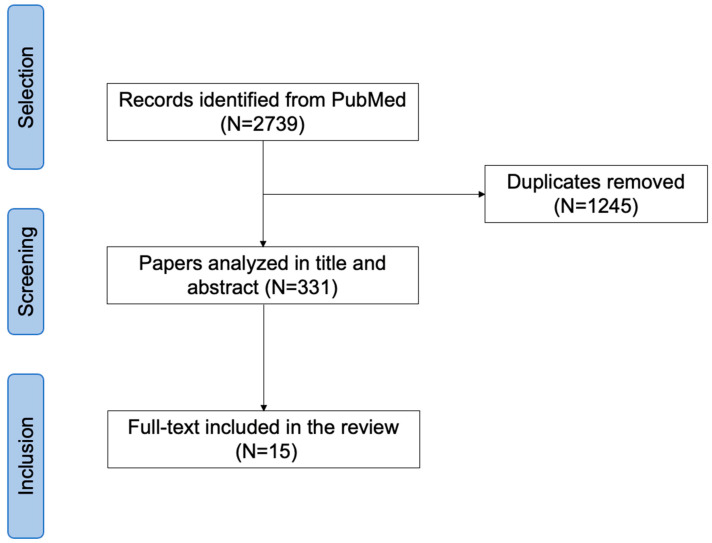
PRISMA flow chart.

2739 articles were identified; after removing duplicates, 331 articles were analyzed. After full-text analysis, 316 papers were excluded due to: lack of data on the effect of lurasidone on depressive symptoms; lack of pre-post assessment; and non-original data. Fifteen papers were finally included in the review (Figure 1).

In terms of study design, three case reports and one case series were found, while the remaining papers were either randomized controlled trials [33,34] or post hoc analyses [35,36,37] (Table 1 and Table 2). Patient sample sizes ranged from one patient [38,39] (in the case reports by Oguchi et al. and Ricci et al.) to 1330 patients [40]. 

In seven experimental studies [34,37,40,41,42,43,44], the efficacy of lurasidone in treating depressive symptoms in patients with schizophrenia has been evaluated against placebo treatment. In the remaining four experimental studies, the efficacy of lurasidone has been compared with risperidone, quetiapine extended releases, or different dosages of lurasidone.

The majority of studies used validated instruments to assess the severity of depressive symptoms in patients with schizophrenia, including the Positive and Negative Syndrome Scale (PANSS), the Montgomery–Åsberg Depression Rating Scale (MADRAS), the Clinical Global Impression–Severity Scale (CGI-S), and the Calgary Depression Rating Scale. Only the study by Oguchi et al. [38] used a non-standardized assessment tool. In addition, most studies also used tools (such as the EQ-5D-3L) to assess quality of life [41] and satisfaction with the pharmacological treatment received (e.g., the Medication Satisfaction Questionnaire) [45].

Lurasidone was effective in improving depressive symptoms reported by patients with schizophrenia in all included studies [40,42,44,45], as well as positive and negative symptoms. In particular, Patel et al. [35] found a significant reduction in the severity of depressive symptoms as assessed by the MADRS at 12-month follow-up.

The efficacy of lurasidone on depressive symptoms has been confirmed at various doses. In particular, in a sample of 244 patients randomized to receive lurasidone 40 mg or lurasidone 80 mg, McEvoy et al. [44] found a significant reduction in depressive symptoms in all randomized groups.

In an open-label study by Correll et al. [42], lurasidone was also effective in improving depressive symptoms over the long term. The open-label phase of the study lasted nearly two years, and patients received flexible doses of lurasidone—ranging from 40 to 120 mg—and reported significant improvements in depressive symptoms.

A recent case report by Ricci et al. [46] highlighted the efficacy of lurasidone in the treatment of first-episode psychosis patients with prevalent depressive symptoms. The authors reported a significant improvement in clinical domains and level of functioning associated with the good tolerability of the drug. Moreover, Ricci et al. [39] described in a case series the use of lurasidone in treating depressive symptoms in patients with schizophrenia and cannabis. In a case series on patients with treatment-resistant schizophrenia receiving clozapine by Olivola et al. [47], all patients received lurasidone as add-on treatment and therefore achieved a significant reduction of both positive and negative symptoms, with no significant adverse effects.

The presence of side effects has been specifically evaluated only in the study by Olivola et al. [47] using the Udvalg for Kliniske Undersøgelser Side Effect Rating Scale (UKU-SERS), while another study included the evaluation of the levels of satisfaction with medication using the Medication Satisfaction Questionnaire [45]. 

## 4. Discussion

Schizophrenia is a severe mental disorder characterized by heterogeneous clinical dimensions, positive and negative symptoms, and disorganized behaviors. The heterogeneous symptom constellations involve cognitive, behavioral, and emotional symptoms, but no single symptom can be considered pathognomonic for the disorder. For many decades, the presence of depressive symptoms in patients with schizophrenia has been overlooked, although it is a common clinical feature in patients with schizophrenia. In particular, depressive symptoms can cause severe impairment in patients with schizophrenia, not only because they usually increase suicide risk but also because they reduce social functioning and quality of life [48,49].

Therefore, it is essential to address and appropriately treat the heterogeneity of the clinical presentation of patients with schizophrenia. Moreover, patients with schizophrenia present comorbid psychiatric disorders, including the increased prevalence of anxiety, depressive and substance use disorders, and high suicidality rates. Depressive symptoms as well as full-mood episodes are common in schizophrenia but should be present for only a relatively brief period. Depressive symptoms show a modal prevalence of about 25%, and they may occur in all phases of schizophrenia.

Depressive symptoms during a psychotic episode may be related to multiple factors, adding complexity to the diagnostic process. Depressive symptoms in schizophrenia may be induced by several clinical factors, including medication side effects, the presence of physical illnesses, etc.; thus, it is important to highlight the temporal course of the onset of depressive symptoms, either in the prodromal phase (acute dysphoria or depressive symptoms overlapping with negative ones) or during the full-blown illness (i.e., depressive presentations with or without acute psychotic symptoms) [50]. Depressive symptoms seemed to be part of schizophrenia. Patients with schizophrenia—regardless of gender—present depressive symptoms frequently, and these symptoms do not appear to be simply a by-product of age, neuroleptics, family history, negative symptoms, or movement disorder.

Overall, in line with the Kraepelinian dichotomy, depressive symptoms have traditionally been considered nosologically distinct from those of schizophrenia, although recent evidence tends to challenge such assumptions by demonstrating the potential limitations of a strictly categorical approach [51]. Recently, the need for a more dimensional approach has been argued, as depression in schizophrenia is not only an artifactual comorbidity but may be a real element of psychotic symptomatology, as clinical patterns may emerge with different hierarchical orders [52].

However, the management of depressive symptoms in patients with schizophrenia is currently an area of concern, particularly in relation to their pharmacological treatment [53]. In the Supplementary Materials, two clinical cases based on ordinary clinical practice have been reported as an example of using lurasidone for managing depressive symptoms in patients with schizophrenia (Supplementary Materials Table S1).

Vakalopoulos and Fitzroy [54] proposed a new nosological approach to psychosis based on the pharmacological differences associated with depressive and negative symptom dimensions (again based on a relative monoaminergic-muscarinic imbalance in signaling). Dondé et al. [55] critically reviewed recent international guidelines for the treatment of depressive symptoms in people with schizophrenia, highlighting the need for more specific and targeted approaches that take into account the complex interplay of different symptom clusters. Mulholland and Cooper [56] have previously highlighted the importance of treating depressive symptoms in the context of schizophrenia with specific pharmacological agents, emphasizing their crucial impact on overall functioning and well-being. In a recent meta-analysis, Gregory et al. [57] concluded that despite the therapeutic potential of antidepressants for the treatment of depression in schizophrenia, the currently available literature does not clearly confirm the usefulness of this strategy.

Lurasidone showed specific activity in improving depressive symptoms in people with schizophrenia [43]. This is an important finding, as depressive symptoms are often difficult to manage in people with schizophrenia and can have a significant impact on their overall well-being. It is noteworthy that lurasidone appears to be effective not only in treating depressive symptoms but also in alleviating both positive and negative symptoms associated with schizophrenia. This broader effect on the range of symptoms is a positive feature, as it suggests that the drug may provide holistic benefits to individuals with this complex disorder. McEvoy et al.’s [44] finding that different doses of lurasidone, specifically 40 mg and 80 mg, were effective in reducing depressive symptoms suggests that the drug’s efficacy may be dose-dependent and may guide clinicians in tailoring treatment to individual patient needs. In addition, the efficacy of lurasidone in treating patients with first-episode psychosis who present with predominant depressive symptoms suggests that lurasidone may be a valuable treatment option not only for established cases of schizophrenia but also for individuals in the early stages of the illness. The good tolerability of lurasidone is an important factor that may positively influence treatment decisions.

In four studies, the efficacy of lurasidone in treating depressive symptoms has been compared with risperidone or quetiapine treatment, showing the superiority of lurasidone treatment. These data are very relevant since risperidone has been specifically evaluated in several studies for improving depressive symptoms in patients with schizophrenia [58,59]. In particular, patients receiving risperidone—compared with those treated with haloperidol—reported a significant reduction in depressive symptoms. Therefore, it is extremely important to have found that lurasidone—an innovative third-generation antipsychotic—is effective in treating depressive symptoms in patients with schizophrenia. Such data are particularly relevant since lurasidone has a safe tolerability profile and is well perceived by patients [29,30,31,32]. However, in the future, it should be of interest to conduct real-world studies aiming to assess and compare the effectiveness of various third-generation antipsychotics (including brexpiprazole and cariprazine) in ordinary clinical practice. It was out of the scope of the present review to include and evaluate studies considering the impact of other antipsychotics on affective symptoms in patients with schizophrenia.

Moreover, another important finding—which deserves further rigorous studies—is related to the use of lurasidone for managing people with treatment-resistant schizophrenia receiving clozapine. Evidence for using lurasidone for managing depressive symptoms in such a complex target group of patients with schizophrenia is quite limited, based on the case series published by Olivola et al. [47]. However, it is necessary to highlight that up to 30% of patients with schizophrenia experience minimal to no symptomatic response from antipsychotic treatment. The only approved pharmacotherapy for treatment-resistant schizophrenia is clozapine, which must be carefully managed due to its side effects and safety concerns. Furthermore, half of patients with treatment-resistant schizophrenia are intolerant or resistant to clozapine. It appears clearly that using innovative antipsychotic drugs, such as lurasidone, the complex management plan of patients can be personalized and should be useful to improve the long-term prognosis of affected patients. Furthermore, lurasidone is effective in treating depressive symptoms in cannabis-induced psychotic disorders. Among psychoactive substances, cannabis represented the most commonly used ones worldwide, with one in four adults reporting to have assumed cannabis at least once during their lifetime. Several reasons have been identified for using cannabis, such as the effects of relaxation, euphoria, and sociability. However, several studies have confirmed that cannabis use is associated, in the long term, with the risk of developing full-blown psychosis. The documented psychotomimetic effects are largely attributable to THC, the main psychoactive compound in cannabis, which acts on the central nervous system by primarily binding to the CB_1_ cannabinoid receptors. The availability in the illegal market of high-potency forms of cannabis has represented a growing concern for the potential adverse effects on mental health. Cannabis use is related to an earlier onset of psychotic illness, and a psychotic breakdown may occur almost three years earlier in cannabis users compared to non-users. Cannabis use may influence the expression of prodromal symptoms and progression to psychosis in individuals at high risk due to the complex interplay among personal, genetic, and social variables, defining the individual’s liability to develop a full-blown psychotic disorder. The incidence of cannabis-induced psychotic disorders is estimated to be 2.7 per 100,000 person-years, with a conversion rate to schizophrenia-spectrum disorders ranging between one-third and one-half. Cannabis-user patients developing schizophrenia disorders report higher levels of positive and negative symptoms compared to those not using cannabis. Moreover, people developing schizophrenia following the use of cannabis have a worse long-term outcome in terms of days of hospitalization, adherence and compliance to pharmacological treatment, number of relapses, and levels of functioning. The management plan of patients with cannabis-induced psychosis and schizophrenia has not been clarified yet by guidelines, and there is no specific treatment with a clear superiority to another. Therefore, the case report by Ricci et al. [39] reporting the efficacy of lurasidone treatment in reducing the severity of both positive and depressive symptoms in patients with cannabis-induced psychosis is very relevant and encouraging. However, such data must be complemented by evidence coming from more structured and rigorous controlled-randomized trials.

Only two studies specifically evaluated safety, tolerability, and patients’ satisfaction with the medication, confirming that lurasidone has a good tolerability and safety profile. In particular, the principal advantages of lurasidone over some other antipsychotics are its highly favorable metabolic profile and once-daily dosing regimen. In the selected studies, patients did not report any significant side effects, which should have improved adherence to treatment and long-term outcomes in patients with schizophrenia. However, further well-designed studies are needed to confirm the good tolerability profile of lurasidone in treating the depressive symptoms of patients with schizophrenia. Moreover, the final aim of the management plan for patients with schizophrenia is represented not only by the remission of clinical symptoms but also by improving their quality of life and long-term recovery. Therefore, selecting pharmacological treatments with a good safety and tolerability profile is essential for the appropriate management of patients with schizophrenia [60].

The present review has some limitations that need to be acknowledged. First, it was not possible to perform a meta-analytic analysis because of the extreme variation in the assessment tools used to evaluate the presence and severity of depressive symptoms in patients with schizophrenia. The tools included the PANSS or the MADRS, while one study used a non-validated assessment tool. Therefore, the high heterogeneity of such assessment tools, with none specifically designed to assess depressive symptoms in patients with schizophrenia, highlights the need to promote future validation studies of assessment tools tailored to the specific psychopathological features of depressive symptoms reported by patients with schizophrenia. It should be useful to use a multilevel approach, starting with a focus group involving users, caregivers, and experts in the field of psychometric development and schizophrenia research, to identify the most important specific features of depressive symptoms reported by patients with schizophrenia. A subsequent step should include the development of a preliminary new questionnaire to be validated in a large and representative sample of patients with schizophrenia.

Although a systematic search has been performed, it is possible that some trials have been omitted due to restrictive selection criteria or the language of publication, which could have introduced a selection bias.

The limitations of the selected studies should be acknowledged, including the short follow-up period [33,34,40,41,44], the lack of an active comparator [35,36,40,41,42], and the strict patient inclusion criteria. In addition, only one study [42] included patients in an acute phase/exacerbation of schizophrenia, so further real-world studies are needed to expand the generalizability of the findings.

Furthermore, studies are limited regarding the long-term effects of lurasidone on patients with schizophrenia; therefore, there is a need to promote further real-world studies with a longitudinal design and robust methodology in order to fill this gap.

## 5. Conclusions

Depressive symptoms in patients with schizophrenia have traditionally been overlooked and not considered of primary clinical relevance, especially when compared to positive and negative dimensions. A modern clinical approach to schizophrenia requires a personalized treatment plan that takes into account all clinical domains, including the positive, cognitive, negative, and depressive dimensions of psychosis. In addition, the clinical characterization of each individual case requires a detailed assessment of other variables, including the presence of adverse childhood experiences, personality traits, recovery styles, and coping strategies [61]. The selection of the most appropriate pharmacological and non-pharmacological treatments, targeting the different clinical domains, should be based on a careful characterization of the individual case. Among pharmacological treatments, lurasidone seems to be particularly effective for the depressive symptomatology of schizophrenia.

The neglected treatment of depressive symptoms, the persistence of residual symptoms, and the adoption of unhealthy lifestyle behaviors represent some of the most relevant clinical unmet needs in the management of patients with schizophrenia [62,63]. The dichotomy between psychotic and depressive dimensions could be overcome by adopting a transnosographic approach to mental disorders [21]. For example, Demyttenaere et al. [64] found an association between depressive symptoms and other clinical dimensions in schizophrenia and highlighted the need for more tailored intervention efforts across mood and psychotic disorders. There is a need to dedicate more attention to the needs of patients with schizophrenia, who report high levels of depressive symptoms. In particular, it should be useful to develop innovative assessment tools in order to capture the complexity of such clinical presentations [43,65,66,67,68,69,70].

In conclusion, we believe that lurasidone is a promising treatment option for patients with schizophrenia, including those with depressive symptoms. Lurasidone is effective for positive, negative, and depressive symptoms in patients with schizophrenia, has a positive impact on long-term outcomes, and may be beneficial in the early stages of the illness [71,72,73,74,75].

## Figures and Tables

**Table 1:** Experimental studies included in the narrative review (N = 11).

Author, Year, Country, and Study Design	Sample Size	Assessment Tool(s)	Main Findings	Study’s Limitations
Miura et al., 2022, Japan Double-blind study and 12-week extension study [34].	289 adult patients with schizophrenia.	Positive and Negative Syndrome Scale (PANSS) subscale scores Clinical Global Impression–Severity Scale (CGI-S) The Calgary Depression Scale for Schizophrenia (CDSS)	Patients reported a mean endpoint (week 12) change from the open-label baseline for lurasidone modal 80 mg/d vs. modal 40 mg/d of −0.5 vs. −0.4 at the CGI-S scale and −0.7 vs. −0.1 at the CDSS score. Patients receiving lurasidone modal 80 mg/d reported greater reductions on the PANSS positive subscale (−3.0 vs. −2.3), PANSS negative subscale (−1.9 vs. −1.7), and global scores (−5.1 vs. −3.8) compared to modal 40 mg/d.	Lack of random allocation to different dosages during the extension phase No patients were treated with a fixed dosage of lurasidone 80 mg/d due to the study design Short follow-up period No statistical test for identifying differences between groups
Iyo et al., 2021, Japan A 6-week, double-blind, placebo-controlled study was enrolled in a 12-week open-label extension study with flexible dosing of lurasidone at 40 or 80 mg/day [41].	289 patients were enrolled in the open-label extension study.	Positive and Negative Syndrome Scale (PANSS) total score Clinical Global Impression–Severity Scale (CGI-S) The Calgary Depression Scale for Schizophrenia (CDSS) European Quality of Life 5 Dimensions 3 Level Version (EQ-5D-3L) EuroQol visual analog scale (EQ-VAS)	Treatment with lurasidone compared to placebo has resulted in a broad range of benefits, including improvements in depressive and cognitive symptoms. Patients reported a significant reduction in clinical sympotmatology, scoring −29.4 (17.6) at the PANSS total score in the double blind and −8.8 (13.3) in the open-label study. The CDSS score was stable. An improvement in quality of life evaluated at EQ-5D-3L was found at week 12. Patients reported an overall mean (SD) increase of 0.097 (0.190) (double-blind study) and 0.028 (0.141) (open-label study). An improvement in quality of life evaluated at the EQ VAS was found, scoring 16.8 (24.1) and 5.3 (18.8) at the double-blind and open-label baselines, respectively.	Short follow-up period Lack of a comparator No statistical test for identifying differences between groups
Patel et al., 2020, USA Post hoc analyses [35].	Patients with schizophrenia were randomized to lurasidone (n = 399) and risperidone (n = 190), of whom 129 and 84 continued into OLE, respectively.	Positive and Negative Syndrome Scale (PANSS) Clinical Global Impression–Severity Scale (CGI-S) Montgomery–Asberg Depression Rating Scale (MADRS)	Patients treated with lurasidone (mean change −0.8, 95% CI: −1.6, 0.0) and risperidone (mean change −2.3, 95% CI: −3.2, −1.3) both reported a reduction in the MADRS total score from baseline to month 12. A statistically significant difference was found between patients treated with lurasidone and with risperidone only at month 12 (*p* = 0.013).	Exclusion criteria: patients with a previous poor or inadequate response/intolerability to risperidone or with an acute exacerbation of schizophrenia Open-label design Lack of a control group
Mattingly et al., 2020, USA Post hoc analyses [36].	Of the 236 patients who completed the initial 12-month double-blind study, 223 (94.5%) continued into the open-label extension study.	Positive and Negative Syndrome Scale (PANSS) Clinical Global Impression (CGI) Montgomery–Åsberg Depression Rating Scale (MADRS)	After completion of 12 months of double-blind treatment with lurasidone or risperidone, mean change scores were −1.7 and −2.6, respectively.	Open-label design Lack of randomization Lack of active control group Small sample size in the risperidone switch group Exclusion criteria: patients with an acute exacerbation No statistical test for identifying differences between groups
Feng et al., 2020, China Randomized, flexible-dose, double-blind, double-dummy, 6-week non-inferiority study comparing the efficacy and the safety of lurasidone to risperidone [33].	444 patients were screened to obtain an intent-to-treat sample of 384 patients, of whom 54 discontinued treatment prior to 6 weeks.	Positive and Negative Syndrome Scale (PANSS) Positive and Negative Syndrome Scale (PANSS)—positive symptoms subscale Positive and Negative Syndrome Scale (PANSS)—negative symptoms subscale Clinical Global Impression–Severity of Illness (CGI-S) Clinical Global Impression–Improvement scale (CGI-I) The Calgary Depression Scale for Schizophrenia (CDSS) Barnes Akathisia Scale (BAS) Abnormal Involuntary Movement Scale (AIMS) Simpson Angus Scale (SAS)	Patients treated with risperidone compared to lurasidone reported a significant increase in glucose (+1.1 mg/dL vs. −0.3 mg/dL; *p* < 0.05), serum prolactin (+60.4 ng/mL vs. +3.5 ng/mL; *p* < 0.001), and body mass index (+0.45 kg/m^2^ vs. +0.20 kg/m^2^; *p* < 0.05). No significant differences were found at BARNES, AIMS, and SAS between patients treated with lurasidone and those treated with risperidone (+0.2 vs. +0.2, *p* = 0.369; +0.0 vs. +0.0, *p* = 0.922; +0.5 vs. +0.8, *p* = 0.098 at week 6).	Exclusion criteria: patients with physical and psychiatric comorbidities Short follow-up (i.e., 6 weeks)
Harvey et al., 2017, USA Post hoc analysis based on data from a previously randomized, double-blind, six-week placebo- and active controlled acute study, followed by a one-year [37].	488 patients with schizophrenia, were randomly assigned to lurasidone 80 mg/d, lurasidone 160 mg/d, quetiapine XR 600 mg/die, or placebo.	Item G12: “impaired insight and judgment” of the Positive and Negative Syndrome Scale (PANSS) Montgomery–Åsberg Depression Rating Scale (MADRS) Quality of Well-Being Scale Self-Administered (QWB-SA) scale	Patients treated with lurasidone reported a significant improvement in “insight and judgment” from acute phase baseline to week 6 (effect size = 0.61 for 160 mg/d vs. placebo, *p* < 0.001; effect size = 0.58 for 80 mg/d vs. placebo, *p* < 0.001), as well as those treated with quetiapine XR 600 mg/d (effect size = 0.67 vs. placebo, *p* < 0.001) compared to patients treated with placebo.	Using the single PANSS-item G12 for measuring insight and judgment
Correll et al., 2016, USA Open-label study [42].	A total of 496 patients were randomized in the core acute study. Patients who completed a 6-week, double-blind (DB), placebo-controlled trial continued in a 22-month, open-label (OL) study during which they received once-daily, flexible doses of lurasidone, 40–120 mg.	Positive and Negative Syndrome Scale (PANSS) Clinical Global Impression– Severity Scale (CGI-S) Montgomery–Åsberg Depression Rating Scale (MADRS)	Patients reported a mean change from DB baseline of −43.6 and −28.4 at the PANSS total score at month 24. A significant reduction in MADRS score was found (10.6 ± 6.7 vs. 11.4 ± 6.09). Treatment with lurasidone was associated with a mean change in weight of +0.4 kg at month 12 and +0.8 kg at month 24. Median change to month 12 and month 24, respectively, was −1.0 and −9.0 mg/dL for total cholesterol; 0.0 and −1.0 mg/dL for LDL; +1.0 and −11.0 mg/dL for triglycerides; and 0.0 and −0.1/% for HbA1c.	Lack of randomization Lack of an active comparator Lack of a double-bind design High attrition rate
Nasrallah et al., 2015, USA Patient-level data were pooled from four similarly designed, double-blind, placebo-controlled, 6-week registration studies of lurasidone (40–160 mg/d) [43].	Adult patients with an acute exacerbation of schizophrenia. N = 1330 patients with acute schizophrenia (N = 898 lurasidone, N = 432 placebo).	Montgomery–Åsberg Depression Rating Scale (MADRS)	At week 6, patients treated with both doses of lurasidone reported a significant reduction of depressive symptoms evaluated at MADRS (8.4) compared to those treated with placebo (10.2) (*p* < 0.001).	Differences in study design, study duration, severity of depressive symptoms at baseline, and outcome measures used in the pooled analysis
Citrome et al., 2014, USA Open-label, 6-month study [40].	Of the 198 patients who completed the core 6-week study, 149 (75.3%) entered the extension study. Clinically stable, but symptomatic, outpatients with schizophrenia or schizoaffective disorder were switched to lurasidone.	Positive and Negative Syndrome Scale (PANSS) Clinical Global Impression Severity of Illness (CGI-S) scores Calgary Depression Scale for Schizophrenia (CDSS) scores	Patients reported a significant reduction in PANSS total score (−8.2 ± 12.6), in CGI-S score (−0.39 ± 0.85), and in CDSS score (−1.2 ± 4.3).	Short follow-up period (i.e., 6 months) Open-label design Lack of a parallel control group
McEvoy et al., 2013, USA Open-label, 6-month study [44].	244 patients with schizophrenia, randomly allocated to lurasidone 40/40, lurasidone 40/80, and lurasidone 80/80.	Positive and Negative Syndrome Scale (PANSS) Clinical Global Impression– Severity Scale (CGI-S) Calgary Depression Scale for Schizophrenia (CDSS) scores	Significant reductions in depressive symptoms were similar across all randomized groups (*p* = 0.861).	Short follow-up period (i.e., 6 months)
Loebel et al., 2013, USA Prospective, parallel-group study [45].	Patients with schizophrenia randomly assigned to receive 6 weeks of double-blind treatment with once-daily evening doses of lurasidone (80 mg, 160 mg), QXR (600 mg), or placebo, recently hospitalized for an acute exacerbation of psychotic symptoms.	Positive and Negative Syndrome Scale (PANSS) total and subscale scores Clinical Global Impression Severity of Illness (CGI-S) scores Montgomery–Åsberg Depression Rating Scale (MADRS) Negative Symptom Assessment Scale, subject-rated Medication Satisfaction Questionnaire	Patients treated with both doses of lurasidone and QXR-600 mg reported a significantly greater improvement in depressive symptoms compared with the placebo group at week 6, as assessed with the MADRS. Patients treated with lurasidone 80 mg (−22.2 [1.8]) and 160 mg (−26.5 [1.8]) reported a significant improvement compared with the placebo group (−10.3 [1.8]) (*p* < 0.001). Patients receiving QXR-600 mg reported a greater reduction in PANSS total score compared to placebo (−27.8 [1.8], *p* < 0.001). Variation in the PANSS total score was similar for patients treated with lurasidone 160 mg compared to those treated with QXR-600 mg (−26.5 vs. −27.8; *p* = 1.00).	The use of a fixed-dose design facilitated the assessment of dose–response effects Exclusion criteria: patients with physical comorbidity and using other medications

**Table 2:** Case series/case studies included in the narrative review (N = 4).

Author, Year, and Country	Sample Size	Assessment Tool	Main Findings	Study’s Limitations
Oguchi et al., 2023, Japan [38].	One young male patient with schizophrenia, aged 20 years old.	No specified, qualitative interview	The patient developed post-psychotic depression, including despair, overwhelming loss, humiliation, and suicidal ideation, during treatment with paliperidone. He was switched to lurasidone 40 mg monotherapy.	Case report
Ricci et al., 2023, Italy [46].	One young male patient with schizophrenia, aged 19 years old.	Positive and Negative Syndrome Scale (PANSS) Clinical Global Impression–Severity Scale (CGI-S) Montgomery–Asberg Rating Scale (MADRS)	19-year-old male patient with first-episode psychosis (FEP) and predominant depressive symptoms. Remarkable clinical and functional improvement was observed 3 months after the beginning of lurasidone treatment. The patient’s depressive symptoms disappear with a dramatic reduction of psychotic ones, with good tolerance of the drug and without adverse effects. Lurasidone seems to be a promising treatment option for FEP with predominant depressive symptoms.	Case report
Olivola et al., 2023, Italy [47].	Four patients treated with clozapine who were diagnosed with treatment-resistant schizophrenia.	Positive and Negative Syndrome Scale (PANSS) Udvalg for Kliniske Undersøgelser Side Effect Rating Scale (UKU-SERS)	All patients achieved a significant reduction of both positive and negative symptoms, with no significant adverse effects to be reported.	Case series
Ricci et al., 2022, Italy [39].	Four patients experienced their first cannabis-induced psychotic episode.	Unspecified	Lurasidone also appears to be effective in other symptom domains related to schizophrenia, such as depressive symptoms.	Case series

## Data Availability

Data are available upon request from the corresponding authors.

## Supplementary Materials

The following supporting information can be downloaded at: https://www.mdpi.com/article/10.3390/brainsci14030225/s1, Clinical cases on the use of lurasidone for the treatment of depressive symptoms in patients with schizophrenia. Table S1: Case 1; Table S2: Case 2.
