# Supplementary material for: The Role of Lurasidone in Managing Depressive Symptoms in People with Schizophrenia: A Review
Source: Brain Sci. 2024 Feb 28;14(3):225. doi: 10.3390/brainsci14030225 (PMC10968750; doi:10.3390/brainsci14030225)
Supplement: Supplementary file 1 [file brainsci-14-00225-s001.zip › brainsci-2886296-supplementary.pdf]

Supplementary material – "The Role of Lurasidone in Managing Depressive Symptoms in People with Schizophrenia: A Review"

Clinical cases on the use of lurasidone for the treatment of depressive symptoms in patients with schizophrenia

**Supplementary Table 1. Case 1.**<br>**LF, male 22 years-old**
LF, a 22-year-old male, presented to the emergency department with his parents because of disorganized behavior that had begun three months earlier. The patient's mother reported that almost six months earlier, LF had developed a special interest in unusual activities with progressive withdrawal from social activities.<br>According to the parents, the symptoms began with insomnia, being less talkative than usual, abandonment of usual interests (he used to do sports at least twice a week and suddenly stopped), depressed mood with frequent crying, and anxiety. In the following month, he had strange behavior, such as leaving the house at night to walk around the city without any purpose.<br>During the clinical examination, LF stated that he had been using cannabis regularly (at least twice a week) for the past year and that he had started using cocaine six months ago. He seemed very agitated, tense and anxious, but his speech was suddenly interrupted by crying. LF was therefore admitted to inpatient treatment and diagnosed with schizophrenia because his psychotic symptoms had been present for more than six months and his depressive symptoms had not reached the threshold for a depressive episode. He was started on LUR at 74 mg/day in combination with delorazepam 3 mg/day. The depressive symptoms gradually improved, while the psychotic symptoms decreased

slightly in intensity and he began to criticize them. Lurasidone was increased to 148 mg/d after one week. After 21 days of inpatient treatment, the patient was discharged because both psychotic and depressive symptoms were significantly reduced and his personal functioning improved.

**Supplementary Table 2. Case 2.**<br>**AS, female 58 years-old**
AS, a 58-year-old woman, presented to the general psychiatric outpatient clinic with marked loss of interest in usual activities, depressed mood, hopelessness, anxiety, and sleep disturbances associated with auditory hallucinations. She reported hearing a male voice telling her that her life was worthless and that she could end her life. On clinical examination, AS reported that her mother suffered from schizophrenia. Three years ago, AS was diagnosed with breast cancer (treated with hormone therapy) and is still undergoing cancer follow-up.<br>In the past two years, she has been treated with several antidepressants (e.g., paroxetine, citalopram, mirtazapine, etc.) and antipsychotics (e.g., haloperidol, aripiprazole, and risperidone). She was referred to the outpatient clinic due to incomplete recovery. At referral, AS was treated with risperidone 4 mg/d, escitalopram 20 mg/d, and lorazepam 1 mg/d. Routine laboratory tests revealed hyperprolactinemia (2312 mUI/L). Given the incomplete and inadequate response, persistence of clinical symptoms, and changes in prolactin levels, a switch to lurasidone was proposed. Over the next two weeks, risperidone was gradually tapered and lurasidone was introduced at a daily dosage of 37 mg/, then increased to 74 mg/day. After three months, prolactin levels gradually decreased (to 1261 mUI/L), confirming the small effect of lurasidone on prolactin

metabolism. She continued to take lurasidone with a good clinical response, and prolactin levels were in the normal range after four months.
